# Hemolytic anemia in alcoholic liver disease: Zieve syndrome: A case report and literature review

**DOI:** 10.1097/MD.0000000000008742

**Published:** 2017-11-27

**Authors:** Miao-Xia Liu, Xiao-Yu Wen, Ying-Kit Leung, Yi-Jie Zheng, Mei-Shan Jin, Qing-Long Jin, Jun-Qi Niu

**Affiliations:** a Department of Hepatology, The First Hospital of Jilin University; b Department of Pediatric Gastroenterology, The First Hospital of Jilin University, Changchun; c Medical Scientific Affairs, Abbott Diagnostics Division, Abbott Laboratories, Shanghai, China.

**Keywords:** alcoholic liver disease, case report, hemolytic anemia, jaundice, Zieve syndrome

## Abstract

**Rationale:**

Zieve syndrome, a rarely reported disease resulting from alcohol abuse, consists of a triad of symptoms: hemolytic anemia, cholestatic jaundice, and transient hyperlipidemia. It is largely under-recognized and under-reported, possibly because of unawareness of the condition by physicians. Here, we report a case of Zieve syndrome managed at the Jilin University First Bethune Hospital.

**Patient concerns:**

A 30-year-old Chinese woman presented with a 4-month history of fatigue, yellowish discoloration of the eyes, and tea-colored urine. She had been a heavy drinker for 2 years prior to onset of the disease with an average daily alcohol intake of 60 g/d and more than 80 g/d for the previous 6 months.

**Diagnosis:**

The diagnosis of Zieve syndrome was confirmed based on hemolysis and cholestatic jaundice secondary to alcoholic liver disease and heavy drinking. Bone marrow biopsy and liver biopsy both supported the diagnosis.

**Interventions:**

We treated her with abstinence from alcohol and supportive therapy.

**Outcomes:**

The patient was discharged 14 days after admission with an improvement in symptoms, which continued to subside during the 2-month follow-up period.

**Lessons:**

Doctors confronted with hemolysis in a patient with alcoholic liver disease should be aware of the under-reported Zieve syndrome. Recognition of this syndrome could help doctors avoid unnecessary invasive procedures and emphasize the importance of alcohol abstinence as the mainstay of management. Glucocorticoids may not be useful in treating hemolytic anemia in Zieve syndrome.

## 1 Introduction

Zieve syndrome was first reported by Leslie Zieve in 1958 based on a retrospective study of patients with similar clinical features.^[1]^ The syndrome was not widely accepted by the medical community until Balcerzak published a prospective study and corroborated Zieve's report in 1968. Zieve syndrome is considered to be rare, with about 200 case reports published in the literature since 1958.^[1]^ The pathogenesis of Zieve syndrome remains obscure. Abstinence from alcohol remains the most effective way of treating Zieve syndrome, and most patients will recover within 4 to 6 weeks after alcohol withdrawal.

## 2 Case presentation

A 30-year-old Chinese woman sought medical attention at the Jilin University First Bethune Hospital with complaints of yellowish discoloration of the eyes, dark urine, and fatigue for 4 months prior to admission. The patient had been a heavy drinker for 2 years prior to admission, with an average alcohol intake of 60 g/d and more than 80 g/d over the previous 6 months. The symptoms had progressively worsened since onset of the illness, and she had a history of 2 previous local hospitalizations before being seen in our facility.

### 2.1 First hospitalization

During the patient's first hospitalization, she was diagnosed with hemolytic anemia after some routine tests and examination of her bone marrow. The bone marrow biopsy at her local hospital had demonstrated an obviously active bone marrow proliferation. Profligate, active, nucleated red cells could be seen in a blood smear, and there were signs of hyperplastic anemia, indicating that the anemia was due to hemolysis. At the time, the patient was prescribed a 2-month course of oral prednisolone, but her symptoms worsened after 2 months.

### 2.2 Second hospitalization

The patient's heavy drinking habit persisted even after diagnosis of the illness and her first hospitalization. A second hospitalization occurred 1 month prior to admission at our facility, and it was during this time that the patient was diagnosed with severe acute pancreatitis. A lipid panel also showed serum triglycerides of 311.87 mg/dL. Fortunately, she recovered after treatment at the local hospital. The fatigue and jaundice persisted and worsened nevertheless.

### 2.3 Admission to Jilin University First Bethune Hospital

The patient was then transferred to our hospital for further treatment. She was first admitted to the hematology department of our hospital where a bone marrow biopsy was performed again, the result of which supported the diagnosis of hemolytic anemia but could not explain the hepatic damage and elevated conjugated bilirubin. She was then transferred to the hepatology department.

Physical examination revealed that the patient had scleral icterus. There was facial and conjunctival edema, moon face, and central obesity evident, likely due to the steroid therapy received during her first hospitalization. Her liver could be palpated 6 cm below the right costal margin, and her spleen was palpable 7 cm below the left thoracic cage. There was also mild edema of the lower limbs.

**Figure 1:**
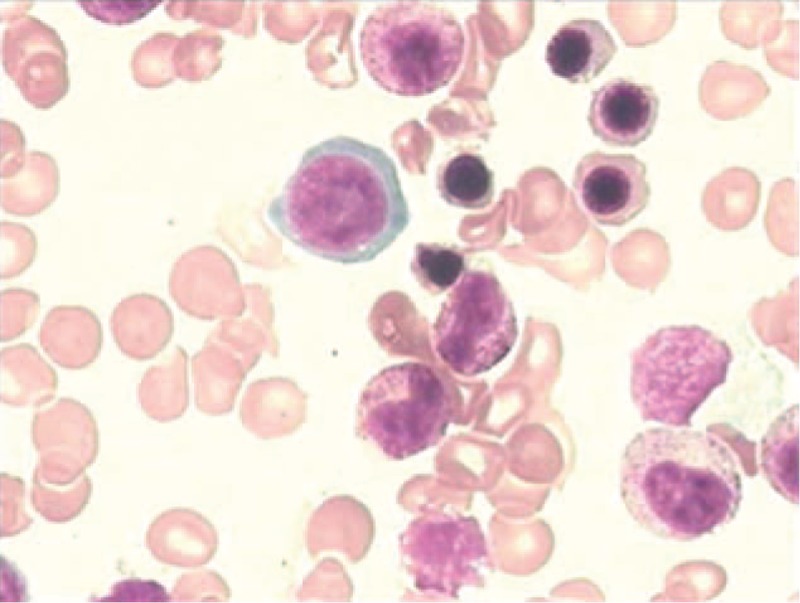
Bone marrow biopsy showing active bone marrow proliferation, mainly due to erythroid hyperplasia. The proportion of granulocytes was normal.

Notable laboratory studies on admission are summarized in Table 1. A peripheral blood smear was compatible with hemolysis and revealed anisocytosis, and an occasional target cell was seen. Hemoglobin electrophoresis was normal, ruling out thalassaemia. An erythrocyte incubation osmotic fragility test was negative, ruling out hereditary spherocytosis. A bone marrow biopsy revealed active bone marrow proliferation of mainly erythroid hyperplasia (Fig. 1). The proportion of granulocytes was normal. The patient's lipid levels tested in the normal or below normal range, in contrast to the elevated triglyceride levels seen during her second hospitalization. Hyperlipidemia in Zieve syndrome can be transient and the patient at that point had been sick for 4 months.

**Table 1:**
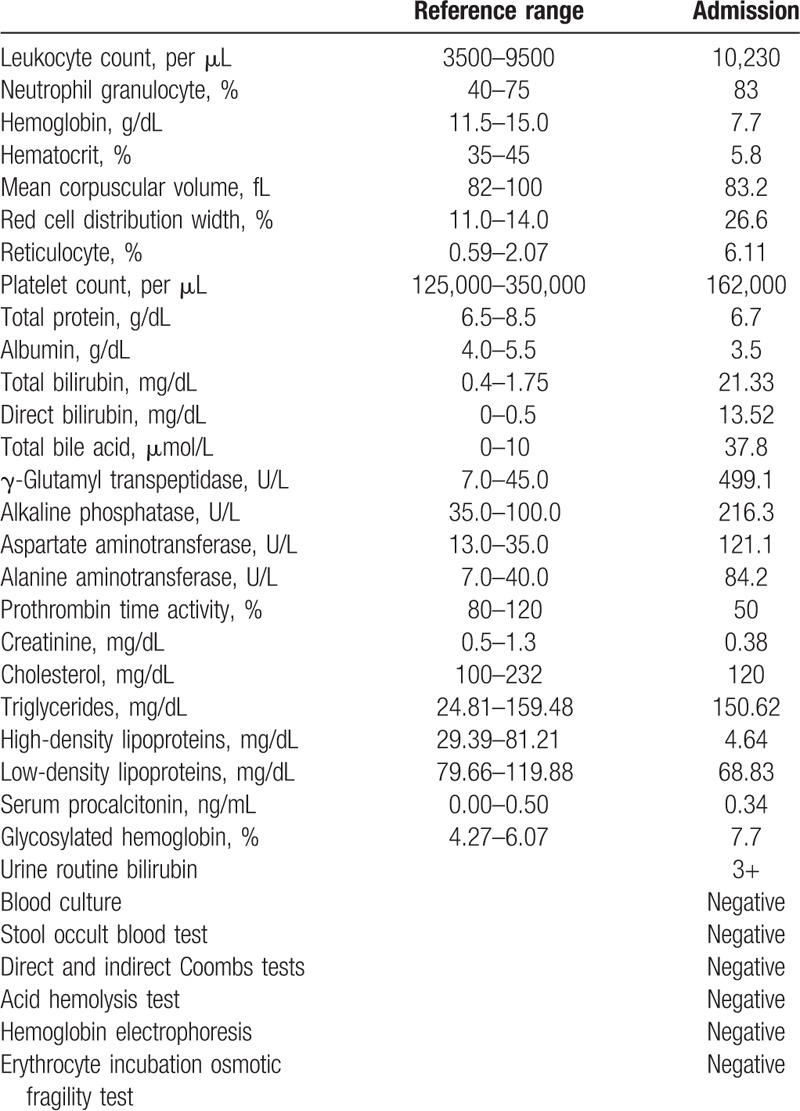
Laboratory data on admission.

An abdominal ultrasound revealed hepatic steatosis. The inferior border of the liver was 5.8 cm below the right costal margin, and the inferior border of the spleen was 7.1 cm below the left thoracic cage. The oblique diameter of the right lobe of the liver was 16.3 cm, and the spleen pachydiameter was 7 cm. The diameters of the portal vein and the splenic vein were 13 and 11 mm, respectively.

**Figure 2:**
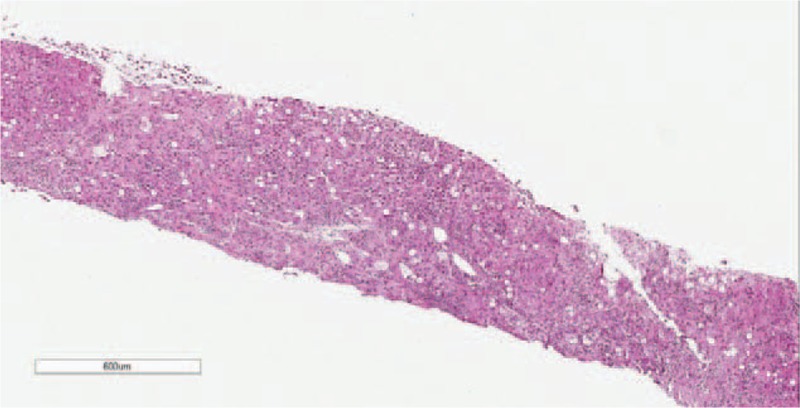
Liver biopsy at low magnification. The liver tissue was badly damaged, and the portal area and central vein were nearly unrecognizable.

**Figure 3:**
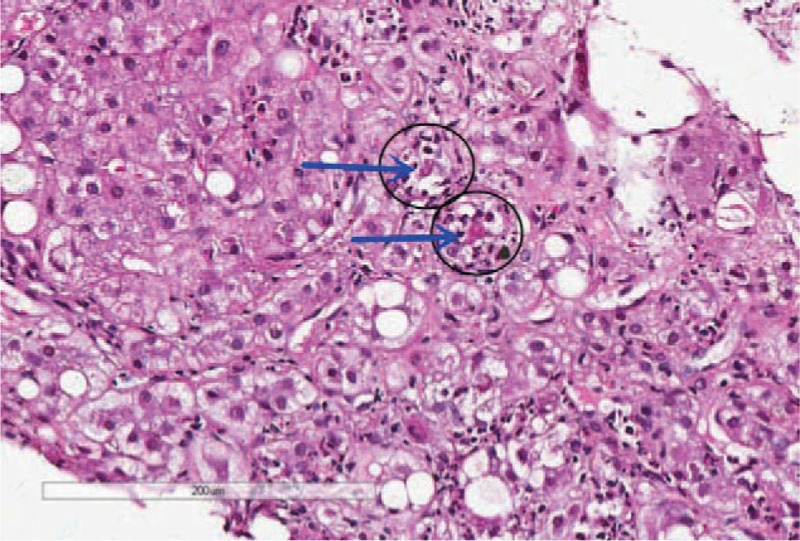
Liver biopsy at high magnification. Mallory body (arrow) surrounded by neutrophils (black circle) and fatty infiltration.

**Figure 4:**
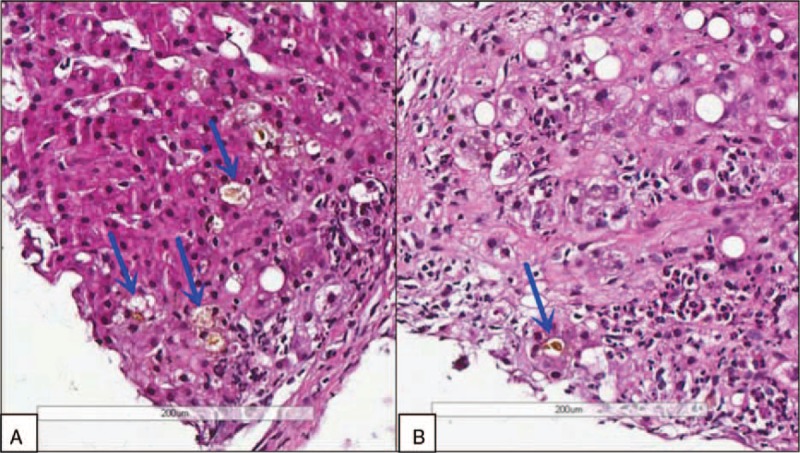
Liver biopsy at high magnification. Cholestasis was seen in hepatocyte (A) and bile canaliculi (B).

**Figure 5:**
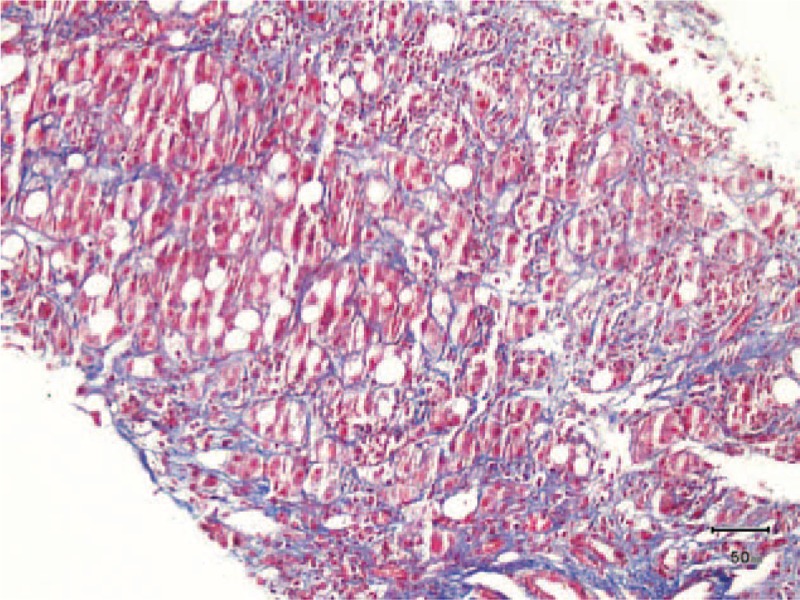
Liver biopsy. Masson staining showing perisinusoidal fibrosis.

Pulmonary computed tomography scanning showed evidence of mild pneumonitis. A liver biopsy revealed mild fatty degeneration, Mallory bodies, and cholestasis (Figs. 2–5). Viral hepatitis, nonhepatotropic viral infection, autoimmune liver disease, and metabolic liver disease were ruled out.

We are of the opinion that this patient had Zieve syndrome based on the combined clinical features of cholestasis, hemolytic anemia, and alcoholic liver disease. Treatment consisted of alcohol abstinence, blood transfusion, and administration of glutathione, diuretics, ursodeoxycholic acid, vitamin supplements, and cefoperazone sodium/tazobactam sodium. Her condition gradually improved, and she was discharged 25 days after admission with orders to completely withdraw from consumption of alcohol and to take oral glutathione, ursodeoxycholic acid, and polysaccharide iron complex for a period of time.

**Figure 6:**
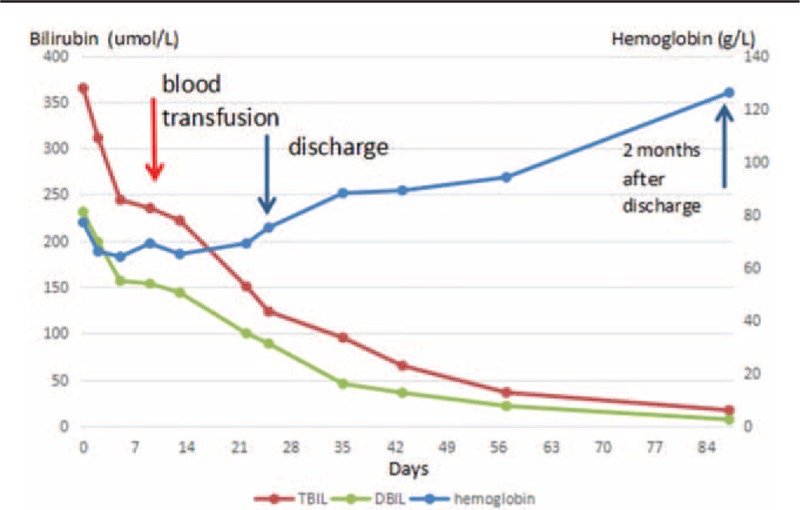
Changes in bilirubin and hemoglobin levels after admission to our hospital.

Two months after discharge, her liver size, portal vein diameter, and levels of blood glucose, hemoglobin, bilirubin, alkaline phosphatase (ALP), and gamma-glutamyltransferase (γ-GT) were almost normalized (Tables 2–4; Fig. 6) at her follow-up. Her spleen pachydiameter dropped from 70 to 56 mm, and the patient's general condition was found to be well.

**Table 2:**
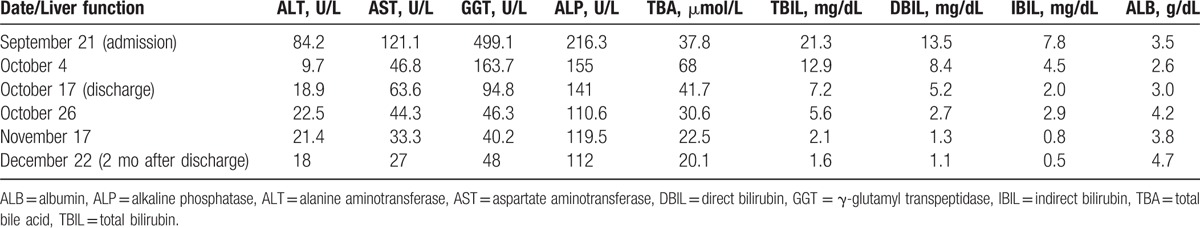
Changes in liver function after admission to our hospital.

**Table 3:**
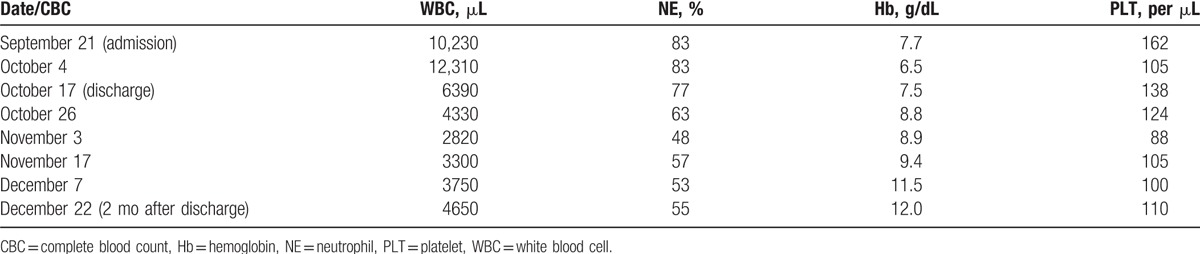
Changes in CBC after admission to our hospital.

**Table 4:**
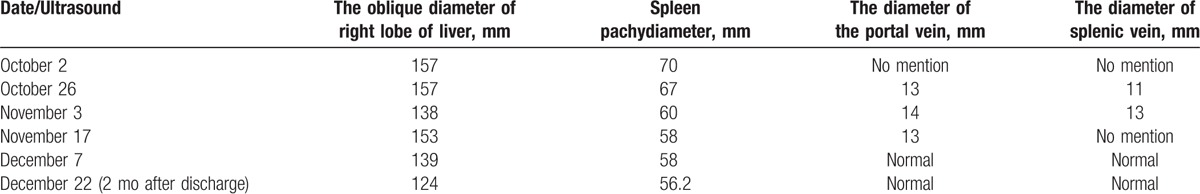
Changes in abdominal ultrasound findings after admission to our hospital.

### 2.4 Literature review

A search of the PubMed database uncovered 120 publications that were related to “Zieve's syndrome” or “Zieve syndrome” after removing duplications and unrelated literature. Among the 120 papers, mostly published by Europeans, 96 were published in non-English languages, and 24 were written in English. The publications were in the form of case reports (47), reviews (5), comparative studies (2), English abstracts (21), letters (6), journal articles (114), and a clinical conference (1). The time periods of publication were as follows: 1958 to 1978 (79), 1978 to 1998 (30), and 1998 to 2017 (11).

## 3 Discussion

The pathogenesis of Zieve syndrome remains obscure but is defined by the triad of jaundice, hyperlipidemia, and hemolytic anemia. The hyperbilirubinemia seen in patients with Zieve syndrome is often profound given the dual contributory mechanisms of hepatocyte injury and hemolysis.^[2]^ Jaundice of this triad is caused by direct bilirubin elevation, which suggests cholestasis caused by liver damage in alcoholism as the main reason instead of hemolytic anemia.

Hyperlipidemia in Zieve syndrome can be transient, as seen in our patient. Suggested mechanisms include an episode of massive mobilization of fat to or from the fatty liver^[3]^ and dysregulated blood lipids due to damaged pancreatic α cells,^[4]^ as alcohol also damages the pancreas.

Zieve suspected that the hemolysis is due to hyperlipidemia and that an abnormal lipid, possibly lysolecithin, may also be present.^[5]^ Some triggering factors, such as lysolecithin and lysocephalin, could markedly aggravate the hemolysis. In addition, alcohol-induced vitamin E deficiency, which reduces polyunsaturated fatty acid levels and causes oxidation of reduced erythrocyte glutathione, leads to enzyme instability and erythrocyte hemolysis.^[6]^ Moreover, Coombs test-negative hemolysis is a salient feature in this triad, which may indicate it is less likely to be an autoimmune hemolytic anemia and would be insensitive to glucocorticoid therapy. Overtreatment of Zieve syndrome with steroids, like our patient experienced during her first hospitalization, may lead to many unnecessary complications.^[7]^

Our patient's diagnosis was established based on a history of heavy drinking, the clinical triad, and other pertinent physical and lab examinations. The patient had a history of alcohol abuse and displayed the triad of jaundice, hyperlipidemia, and hemolytic anemia. Her laboratory examination also supported our diagnosis. Direct bilirubin, ALP, and γ-GT were elevated more markedly than aspartate aminotransferase/alanine aminotransferase. The liver biopsy showed that cholestasis existed in the hepatocyte and bile canaliculi (Fig. 4), suggesting cholestatic jaundice. The liver biopsy also demonstrated steatosis, perisinusoidal fibrosis (Fig. 5), and Mallory corpuscles (Fig. 3), which are features of alcohol liver disease. The peripheral blood smear and bone marrow biopsy demonstrated reticulocytosis, and a negative Coombs test supported the diagnosis of Coombs test-negative hemolytic anemia. The patient also experienced transient hyperlipidemia. Subsequent to her abstinence from alcohol and the initiation of other supportive treatment, the patient recovered gradually. Based on the above findings, the diagnosis of Zieve syndrome was established.

Interestingly, the patient also suffered from severe acute pancreatitis, which may be related to Zieve syndrome. The patient had resumed drinking alcohol after her first hospitalization and diagnosis of hemolytic anemia, and afterward the anemia and jaundice became more serious. She then developed acute pancreatitis. Some reports suggest that pancreatitis is an essential feature of Zieve syndrome,^[8,9]^ with possible mechanisms including an increase in lysophosphatide, which could lead to pancreatitis.

Zieve syndrome is a special type of alcoholic hepatitis and is usually recognized by its associated features: the clinical triad of hemolytic anemia, alcoholic fatty liver, and unexplained jaundice. Unfortunately, this association may not be recognized by most physicians. When diagnosed with Zieve syndrome, most patients will recover 4 to 6 weeks after alcohol withdrawal. Continuing to drink alcohol, however, may aggravate the illness and potentially prove to be fatal.

The critical feature that distinguishes Zieve syndrome from acute alcoholic hepatitis is hemolytic anemia, the hallmark of Zieve syndrome. The differential diagnosis between alcohol-related anemia and Zieve syndrome can be seen in Table 5. The prognosis of the 2 disorders is different: 40% of patients with acute alcoholic hepatitis may progress to liver failure and even death, while in Zieve syndrome, abstaining from alcohol would result in a resolution of the symptoms.^[10]^ Patients with Zieve syndrome may have a higher alcoholic hepatitis score, and this could potentially lead to overtreatment with steroids, inducing immunosuppression and possibly leading to further complications.^[7]^

**Table 5:**
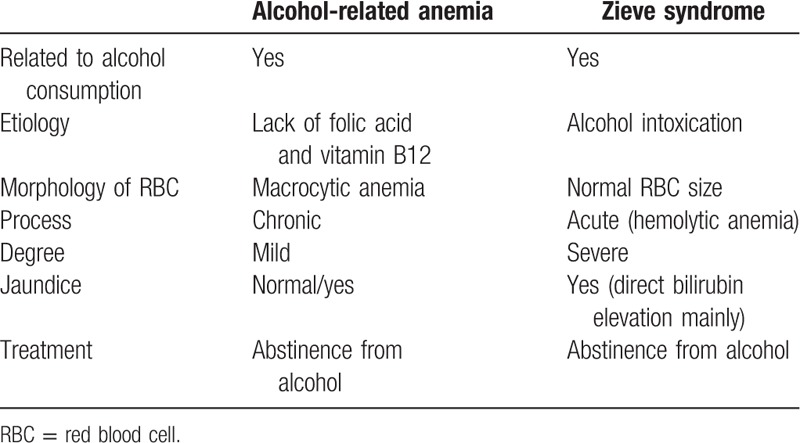
Differential diagnosis between alcohol-related anemia and Zieve syndrome.

Early recognition and diagnosis of Zieve syndrome could avoid unnecessary invasive examinations^[11]^ and treatment. Patients with Zieve syndrome will often exhibit right upper quadrant pain, without acute cholecystitis or bile duct obstruction. Articles have reported that patients with severe jaundice and abdominal discomfort were planned for surgery, but with the eventual recognition of Zieve syndrome as the primary cause of their illnesses, the invasive procedures were avoided.^[12,13]^ For Zieve syndrome, abstinence from alcohol is the most effective treatment. Glucocorticoids, most useful for autoimmune hemolytic anemia, may not be useful for treating Zieve syndrome and may in fact lead to further complications.^[7]^

## 4 Conclusions

Zieve syndrome should be more readily recognized by physicians and the medical community. Best accomplished by increasing medical education, this would enable providers to make the correct diagnosis and perform proper treatment. We anticipate that with the growing consumption of alcoholic beverages in China, there will be more patients afflicted with Zieve syndrome in the future. The early diagnosis and treatment of such patients is of paramount importance to their management. There is a real need to report and increase the body of published English language medical literature describing Zieve syndrome, as most of the present literature is written in non-English languages. Such a spread of English-based medical reporting would more widely circulate necessary knowledge of the disorder and aid in the progress of treating and ameliorating this disease.

## Acknowledgment

The authors thank to our patient, who gave her informed consent for publication.
